# The patient’s first point of contact (PINPOINT) – protocol of a prospective multicenter study of communication and decision-making during patient assessments by primary care registered nurses

**DOI:** 10.1186/s12875-023-02208-0

**Published:** 2023-11-29

**Authors:** Annelie J Sundler, Lena Hedén, Inger K Holmström, Sandra van Dulmen, Karin Bergman, Sofia Östensson, Malin Östman

**Affiliations:** 1 Faculty of Caring Science, Work Life and Social Welfare, University of Borås, Borås, SE-501 90 Sweden; 2 School of Health, Care and Social Welfare, Mälardalen University, Västerås, Sweden; 3 Department of Public Health and Caring Sciences, Uppsala University, Uppsala, Sweden; 4 Nivel (Netherlands Institute for Health Services Research), Utrecht, the Netherlands; 5 Radboud Institute for Health Sciences, Department of Primary and Community Care, Radboud University Medical Center, Nijmegen, the Netherlands; 6 Region Västra Götaland, Närhälsan Källstorp Healthcare Centre, Trollhättan, Sweden; 7 Education, Development & Innovation, Primary Health Care, Region Västra Götaland, Research, Sweden; 8 General Practice/Family Medicine, School of Public Health and Community Medicine, Institute of Medicine, Sahlgrenska Academy, University of Gothenburg, Gothenburg, Sweden

**Keywords:** Communication, Decision-making, Nurse, Nurse-patient interaction, Primary care, Study protocol

## Abstract

**Background:**

A major challenge for primary care is to set priorities and balance demands with available resources. The registered nurses in this study are practice nurses working in primary care offices, playing a large role in initial assessments. The overall objective of this research is to investigate practices of communication and decision-making during nurses’ initial assessment of patients’ health problems in primary care, examine working mechanisms in good practices and develop feasible solutions.

**Methods:**

Project PINPOINT aims for a prospective multicenter study using various methods for data collection and analysis. A purposive sample of 150 patient‒nurse consultations, including 30 nurses and 150 patients, will be recruited at primary care centers in three different geographic areas of southwest Sweden. The study will report on outcomes of communication practices in relation to patient-reported expectations and experiences, communication processes and patient involvement, assessment and decision-making, related priorities and value conflicts with data from patient questionnaires, audio-recorded real-time communication, and reflective interviews with nurses.

**Discussion:**

This research will contribute to the knowledge needed for the guidance of first-line decision-making processes to best meet patient and public health needs. This knowledge is necessary for the development of assessments and decisions to be better aligned to patients and to set priorities. Insights from this research can empower patients and service providers and help understand and enhance feasible person-centered communication strategies tailored to patients’ level of health literacy. More specifically, this research will contribute to knowledge that can strengthen nurses’ communication, assessments, and clinical decision-making in primary care. In the long term, this will contribute to how the competencies of practice nurses and other professionals are organized and carried out to make the best use of the resources within primary care.

**Trial registration:**

ClinicalTrials.gov Identifier: NCT06067672.

## Background

One of the core functions of primary care is first contact accessibility to healthcare. In Sweden, over 36 million health visits are made annually in primary care, and more than 10 million of these are visits to registered nurses (RNs), i.e., practice nurses working in primary care offices [1]. These nurses play a large role in initial assessments and are usually the first primary care contact for the patient. The initial assessment in patient-RN encounters is a prerequisite for subsequent actions that need to be taken so that the right patient ends up at the right time in the right place. Correctly performed, the assessment safeguards the quality of care and decreases preventable healthcare costs, hospitalization and visits to the emergency department [2, 3]. However, nursing practice on assessments and decision-making processes in primary care has received limited attention in research. As a consequence, there is a lack of knowledge of the working mechanisms and processes related to RNs’ communication with patients and assessments. Disseminating and implementing good RN practices will contribute to making primary care fit for the future.

Currently, healthcare is under increasing pressure as a result of shortages in financial and human resources, a rapidly aging population with more long-term conditions and multimorbidity needing primary care [4]. RNs have a prominent role and could contribute considerably to ensuring equal and person-centered primary care and the delivery of sustainable care [5, 6]. Nursing consultations have been shown to achieve similar or better health outcomes in primary care when compared to physicians [7] at lower costs. Optimizing RNs’ role and responsibility could therefore improve primary care services.

In this research, we focus on patients’ first point of contact, i.e., persons seeking primary care for a new health concern or a new episode of a problem [8], different from more continuous primary care over time. Given the variety of patients of all ages and their health complexities, assessments and priorities are demanding. A correct assessment is needed for RNs to make decisions and priorities meeting the urgency and severity of the patient’s health condition. Consequently, decision-making and priority setting can be complex and may have negative consequences for quality of care [9, 10]. This complexity results from conflicting goals, restricted access to services and waiting lists, profitability, and the need to safeguard high-quality care, patient safety and person-centredness. All these circumstances may jeopardize the healthcare system and healthcare professionals’ performance [11].

RN’s communication with the patient is the basis for the exploration and understanding of patient health concerns [12]. It also has therapeutic value, as it can lead to better health outcomes for patients, more effective processing of information, improved adherence to treatment and advice, and higher patient satisfaction [13–15]. Communication is fundamental for sharing expertise, building a trustful relationship and decision-making [16]. In contrast, instances of miscommunication can be linked to problems such as impaired patient safety and poor patient experiences [14, 17]. Moreover, communication with patients can improve the level of health literacy among the public, a rarely noticed part of RNs’ communication practices. Limited health literacy is associated with poor health outcomes and problematic use of healthcare services. Patients with higher levels of health literacy report better health behaviors [18], and efforts to improve health communication among healthcare providers are hence needed [19].

Moreover, communication enhancing patients’ involvement in decision-making can empower them to become active and capable in managing their own health [14, 20, 21]. Ideally, communication should be tailored to patients’ needs and expectations [22, 23]. If not, this may result in dissatisfied patients and more hospital or emergency care visits [23, 24]. A recent review study points out that there are relatively few studies on RNs’ communication practices compared to the number of studies and reviews on physician–patient communication [25]. The nursing studies predominantly focused on nurses’ provision of self-management support and patient education [26–29]. Nevertheless, enhancing RN-patient communication and decision-making in primary care can have far-reaching merits, and providing a solid research base for best practices is hence needed. This paper reports on a study protocol for such research.

### AIM

The overall objective of this research is to investigate current practices of communication and decision-making during RNs’ initial assessment of patients’ health problems in primary care, examine working mechanisms within good practices, and develop innovative and feasible solutions for an integrated care model with RNs in primary care.

The specific aims are as follows:

1. To investigate patients’ expectations and experiences with communication and decision-making during their first contact with an RN in primary care.
2. To investigate patient-RN communication on the level of patient involvement.
3. To investigate RNs’ actual communication, assessments and decision-making, value conflicts and the challenges and strategies they use in prioritizing.
4. To analyze the underlying working mechanisms of good communication practices.
5. To develop methodologies for facilitating efficient processes in assessing, managing, and prioritizing patients in primary care for RNs.

## Methods and analysis

### Design

This is a prospective multicenter study. Varying methods for data collection and analysis will be used to explore communication practices of patient-RN communication in primary care. The STROBE guidelines for the conduct and dissemination of observational cross-sectional studies will be used [30].

### Recruitment procedure

The study aims to collect a purposive sample of 150 patient-RN consultations, including 30 RNs and 150 patients, distributed among the RNs. Patients and RNs will be recruited at primary care centers in three geographic areas of southwest Sweden to include primary care centers in both urban and rural areas and both public and private (under contract) providers. To facilitate recruitment of participants, some of the researchers will act as local contact persons. Initially, RNs will be informed and recruited. Afterwards, the local contact person will be on site to recruit potential patients to minimize the impact on ordinary care work.

First, we will approach eligible RNs. Three researchers, one in each area, will inform RNs about the research and ask for participation. RNs will be recruited after informed consent. Eligible adult patients (aged 18 years or older) with an appointment to the participating RNs during preselected study days will be informed about the research and invited to participate by one of the researchers. Patients who are willing to participate will be asked to give written informed consent.

**Fig. 1:**
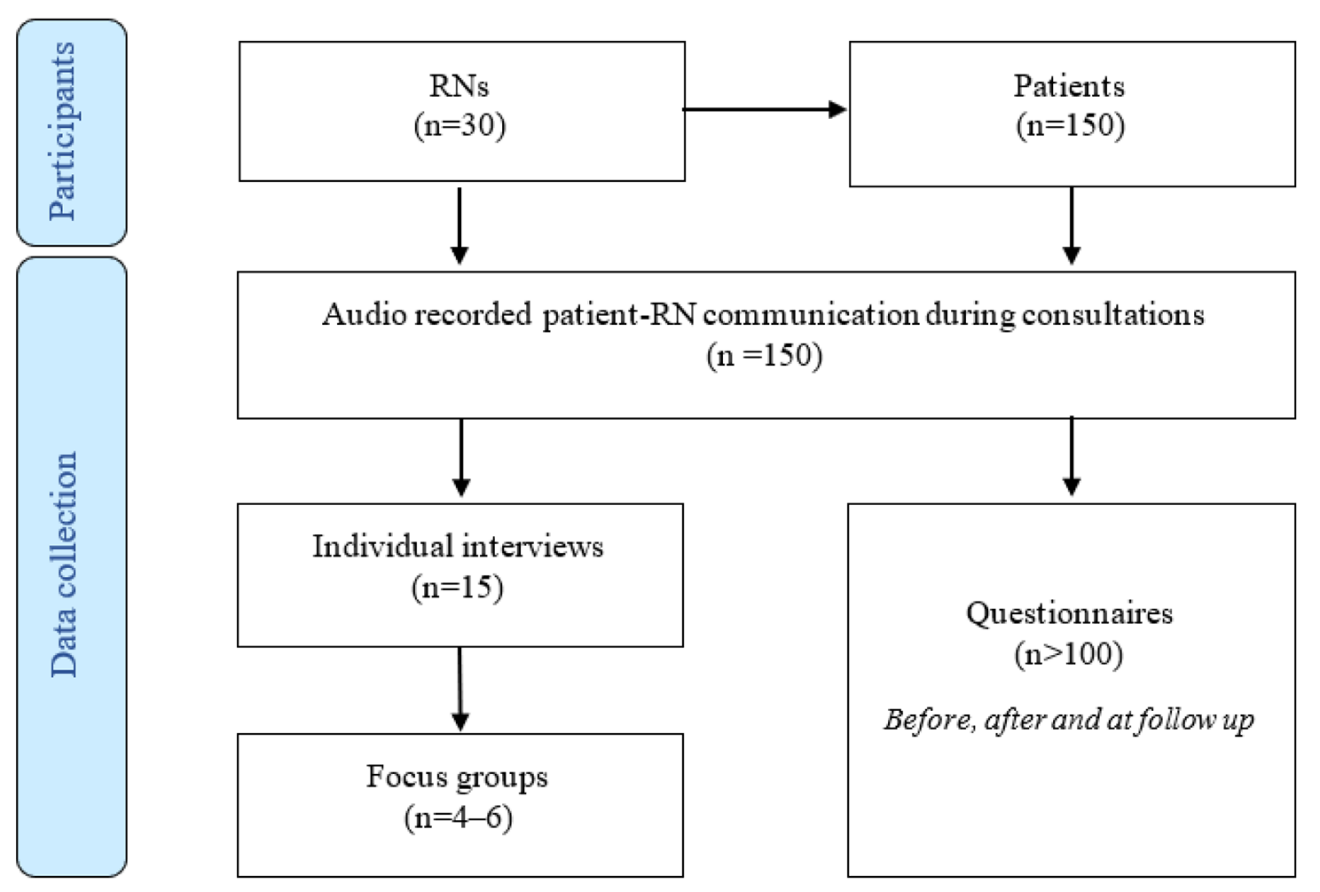
Overview of participants and data

To assess patient-reported outcomes, patients will be asked to complete a questionnaire before and after the consultation and at the two-week follow-up (specific aim 1). To monitor communication during patient-RN visits, audio-recordings of communication during these consultations will be made (specific aim 2). To investigate RNs’ clinical reasoning, assessments made, and priorities or related value conflicts, reflective interviews individually and in focus groups will be conducted (specific aim 3). Data will be collected in 2023 and 2024 and analyzed and reported from 2023 to 2026. For an overview of the participants and data collection, see Fig. 1. The data gathered and results will be used to disentangle working mechanisms using the realist evaluation methodology [31]. This will aid in the development and design of methodologies for working processes that facilitate efficient and person-centered assessments and decision-making for the management and priority of patients in primary care by RNs (specific aims 4 and 5).

### Study size

In conformity with the explorative design, a sample size calculation was not performed, as we did not focus on a single outcome to base our power calculation on in this multiple outcome prospective study. Considering the lack of research in this area, the number of observations of patient-RN consultations was deemed sufficient and feasible, and 5 consultations per RN was considered sufficient to determine their communication style. The number of RNs was also chosen considering that a sample size of 30 can be enough to trust the confidence interval. A two-sided α of 0.05 and 95% power will be considered in the data analysis comparing patient and RN age, sex, and educational level.

### Outcome measures

The outcomes are communication practices of first patient-RN encounters in primary care in relation to:

- Patient-reported expectations and experiences (Questionaries).
- Communication processes and patient involvement (Audio recordings).
- Assessment and decision-making processes and related priorities and value conflicts (Interviews).

**Fig. 2:**
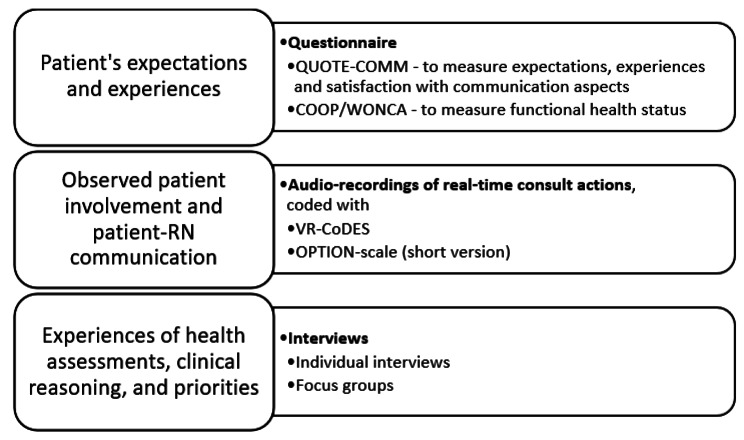
Overview of outcomes related to data sources

For an overview of outcomes related to the respective data to be collected, see Fig. 2.

As the need for and experiences of communication and decision-making are likely to be influenced by patient-related factors, specific attention will be given to the mediating role of patients’ sex, age and level of education and the nature of their health problems.

### Data collection

#### Patient questionnaires

The patient-reported data on expectations and experiences will be gathered at three different time points designed to meet the patients’ experiences prior to the consultation, immediately after, and two weeks after an RN visit. The previsit questionnaire assesses demographics (year of birth, sex, native language and current health problem and the reason for encounter), patients’ expectations of the visit using the QUOTE-COMM (expectations) (QUality Of care Through the patients’ Eyes) [32, 33], and their functional health status using the COOP/WONCA [34, 35]. The postvisit questionnaire includes the QUOTE-COMM (experiences), satisfaction with the consultation and experienced quality of care. The telephone follow-up questionnaire includes the QUOTE-COMM, functional health status using the COOP/WONCA, satisfaction and quality of care.

*The QUOTE-COMM* measures patients’ expectations and experiences with different communication aspects and has been found to be feasible and useful [33]. A total of 19 items, related to three scales, are scored on a 4-point Likert scale. The three scales are a task-oriented scale (6 items) on different medical content, an affect-oriented scale (7 items) on emotional aspects of the interaction, and a therapy-oriented scale (6 items) on therapeutic aspects. Before the consultation, patients score the importance of different communication aspects, e.g., their expectations. After the consultation, patients score the RNs’ performance related to the communication aspects, e.g., their experiences. At telephone follow-up, patients will score their satisfaction with the communication aspects.

*The COOP/WONCA* measures a person’s functional health status on six charts related to six aspects of functioning: physical fitness, feelings, daily activities, social activities, change in health, and overall health. These aspects are rated on a five-point ordinal scale ranging from 1 (better/no limitation at all) to 5 (worse/severely limited), with reference to the last two weeks. Each aspect is reported on a single chart that has a title, a question and a response scale illustrated with easily understandable drawings.

For the assessment of experiences of overall satisfaction and quality of care, a numeric rating scale is used ranging from 1 (very dissatisfied/low quality of care) to 10 (extremely satisfied, very high quality of care). Patients will respond to two questions on (1) their overall experience of satisfaction with the consultation and (2) their experience of quality of care of their consultation.

#### Audio-recordings (ARs)

Observations of real-time consultations will be made with data collected on AR to explore practices of patient-RN communication on health concerns and needs and patient involvement. ARs are coded with OPTION^5^, observing patient involvement in decision-making [36, 37] and the Verona Coding definition of emotional sequences (VR-CoDES) [38, 39]. The ARs will be made by the RNs after receiving information about how to manage these recordings. No researchers will be present during consultations to minimize the impact of the research on the visit. ARs provide powerful, real-life data that allow for analysis of communication and interaction between patients and RNs in their natural context. Analysis of such empirical data on real-time consultations can provide new insights into RNs’ communication practices, in contrast to interviews where there might be gaps between what people report and what they can recall from a situation [40].

#### Interviews

Individual interviews and focus groups with RNs will be used for data collection. All interviews will be audio-recorded and transcribed verbatim. The interviews will be performed during RNs working hours at the primary care centers.

To explore RNs’ clinical reasoning in prioritizing the needs of the individual patient, data will be collected with individual qualitative interviews using stimulated recalls. A purposive sample of the nurses, e.g., 15, will be selected for the interviews with various characteristics of the RNs, their patients, and the consultation. These interviews will be conducted 2–4 weeks after consultation. Each RN will be asked to respond to and elaborate on their conversation with the patient and their clinical reasoning and assessment made during one of their previous consultations during these interviews. Sequences from the ARs will be used to stimulate recall and reflections on their thoughts and actions during the consultation. The RNs will be encouraged to make free and indirect comments about their consultations, while open-ended follow-up questions will be used to obtain as detailed and nuanced descriptions as possible about their clinical reasoning. When needed, the ARs can be stopped. Stimulated recall is a useful method to study consultations [41]. This enables us to further investigate RNs’ communication strategies, their interaction with patients and their clinical reasoning.

To get RNs to reflect and discuss their experiences of clinical reasoning and priorities in patient-RN consultations, data will be collected using four to six focus groups with a total of 30 RNs. A convenience sample of RNs will be selected. This includes both RNs who have previously participated in the research and RNs who have not. Before the interviews, participants will have access to a short text summarizing the findings on RNs assessment from individual interviews. Using focus groups in this way will allow for co-creation and further development of the first analysis from data collected by individual interviews. Four to six RNs will be included in each focus group. The RNs will be asked about challenges and potential conflicts of goals when making assessments and priorities or consequences related to these. Focus groups allow participants to discuss and compare their experiences, which makes it possible to obtain rich descriptions of the phenomenon in focus [42], e.g., their experiences of aspects important for their clinical reasoning and assessments made and challenges and prerequisites for priorities or related value conflicts. A semi-structured interview guide and follow-up questions will be used.

### Analysis and data processing

#### The questionnaire

Descriptive statistics will be used for assessing demographic characteristics and self-reported functional health status (COOP-WONCA). For assessing self-reported levels of different communication aspects (QUOTE-COMM), e.g., before, after and at follow-up, descriptive and inferential statistics will be used.

#### Audio recordings

For analysis of patient involvement, ARs will be coded with the OPTION^5^ scale, a reliable and valid instrument for investigating patient involvement in decision-making [36, 37]. Coding of ARs using OPTION^5^ allows for the assessment of the extent to which RNs involve patients in decision-making. Five items are coded on a 5-point Likert scale, from 0 = ‘zero effort observed’ to 4 = ‘exemplary effort’. The total score is generated by converting the scores to a 0–100 scale and then calculating the average. The higher the score is, the higher the level of patient involvement.

For analysis of patient-RN communication on patients’ expressions on health concerns and RNs’ responses to these concerns, ARs will be coded with the Verona Coding Definitions on Emotional Sequences (VR-CoDES) [38, 39]. The coding identifies episodes and sequences of negative emotions, e.g., elicitations and expressions of patients’ cues and concerns, and RNs’ responses. The VR-CoDES allows for analysis of communication strategies used by RNs to respond to and explore health concerns and needs. When using the VR-CoDES, data are processed and coded directly from the recordings, and transcriptions are not needed. The data will allow for both statistical and qualitative analysis. Descriptive statistics will be used to describe and explore the frequencies and types of health concerns of patients’ concerns and RNs’ responses. The characteristics of emotional sequences of communication and the distribution of categories will be described. In conformity with the data level and distribution, parametric or non-parametric tests will be used for analysis of possible differences between patients’ expressions versus RNs’ responses and sex. There will also be a qualitative analysis of patterns in these sequences related to RNs’ responses and questions used to understand communication strategies efficient for RNs’ exploration and assessment of patients’ health concerns. For statistical analysis, the necessity of performing multilevel analysis will be considered by calculating intraclass correlation coefficients given the nested data structure of consultations and the clustering of data.

For all statistical analyses, SPSS Statistics for Windows, latest version, will be used. The coding with OPTION^5^ and VR-CoDES will be performed by two independent coders, and the inter-rater reliability between them will be checked using Cohen’s kappa [43, 44].

#### Interviews

To explore RNs’ experiences of health assessments, clinical reasoning and priorities, a method for thematic analysis of meanings based on descriptive phenomenology will be used [45]. The analysis will focus on individual RNs’ clinical reasoning and priority, how they engage in these processes, and on sense-making and meanings that emerge in their descriptions. The analysis involves a rigorous process where details and aspects of meanings are explored.

Findings from individual interviews on health assessments and clinical reasoning will be summarized in a short text to use as a facilitator for focus groups. Transcripts of the focus groups will be analyzed with thematic analysis [45]. Themes will be qualitatively derived from an inductive meaning-oriented analysis. Co-coding of interviews will be performed to meet quality criteria for qualitative analysis according to the COREQ guidelines [46].

#### Realist evaluation

Using a realist evaluation (what works for whom in what context), we will disentangle the working mechanisms behind good RN-patient communication practices. We do this by looking for CMO configurations, where C stands for Context (the first point of contact visits in primary care with an RN), M for mechanisms (the parts of the communication process, assessments, and decision-making under study) and O for Outcomes (e.g., meeting patients’ expectations and needs and optimal shared decision-making) [31].

#### Ethical considerations

This research was approved by The Swedish Ethical Review Authority (Dnr Ö24-2023/3.1). We do not anticipate any major risk for the participants in the context of this study. Informed written consent will be obtained from all participants in addition to information about the right to withdraw at any time without consequences. The sampling of participants and data storage, flow, and access will be outlined following legislation and safety routines to safeguard the security, privacy, and confidentiality of participants. All participants will be given information about the study, including how personal data will be processed and about the rights to request information on personal information. Through data management, we will securely organize the data to be available for the researchers involved but inaccessible to unauthorized people. A secure backup of data will be used. Reporting of findings will take place in journals, clinics, professionals and conferences. Research publications and data dissemination will not include identifiable data.

## Time plan and realisation

This research is distributed over four years (2023–2027). The recruitment of participants started in August 2023, and the recruitment and data collection of patient-RN consultations is estimated to take approximately 12 months. These data will be analyzed and reported during 2024 and 2025. The interviews and focus groups will be conducted in 2023 and 2024 to be analyzed and reported between 2024 and 2026.

For realization of the proposed research, an approach involving co-creation and collaboration among practitioners is applied. This means that researchers work in close collaboration with practitioners because they are not only important to facilitate researchers’ access to practices but also because they add value to the scope and focus of research questions, study processes, and the depth and interpretation of the findings. Seminars and workshops will be arranged to stimulate co-creation, validate discussions and ensure the integration of experiences and perspectives from RNs’ practices. Collaboration and co-creation with practitioners from different settings is beneficial for knowledge transfer and learning among researchers and RNs. This is also imperative for research findings to be applied and implemented in clinical practices. This research can generate knowledge applicable for the future development of healthcare provision at the local level.

## Discussion

This research can contribute to the knowledge needed for the guidance of first-line decision-making processes to best meet patient and public health needs. This knowledge is necessary for the development of assessments and decisions to be better aligned to patients and to set priorities. Insights from the research can empower patients and service providers and help understand communication strategies feasible for person-centered care and to support patient health literacy. According to a recent review about RN-patient communication [25], there is an urgent need for in-depth research in this area, especially with the transformation of secondary healthcare to primary care.

The providers’ self-reported ratings of communication skills, empathy, mindfulness and emotional intelligence will give a broad description of traits known to impact communication in healthcare settings. The combination of data from the observational analysis of the visits, rating scales and questionnaires can provide indications of the traits of the healthcare provider that are important to facilitate person-centered communication.

We believe that if assessment processes are optimized properly, RNs can manage uncomplicated acute conditions and illnesses and then free up physicians to more complex patients and how to better meet patients’ demand for help and support. Consequently, this research will contribute to improving primary care services for patients.

A strength of this research is the data collected on real-time communication observed with ARs from patient-RN consultations in its natural context. Naturalistic observations by recordings allow researchers to observe communication between patients and RNs directly in the environment where it occurs in this study without the researcher interacting with participants [47]. The ecological validity in observational studies is considered high.

An important strength of this research is that both patients’ and RNs’ perspectives are in focus combined with data from observed visits, questionnaires and interviews. This will generate rich data that will allow for broad descriptions important for communication and decision-making during RNs’ initial assessment of patients’ health problems in primary care. However, some limitations are worth nothing. First, a constraint of this research may be the use of ARs instead of video recordings. This methodological decision restricts the analysis exclusively to verbal communication, precluding the analysis of nonverbal communication. However, it is worth mentioning that there exists a high correlation between the observations of ARs and video-recordings [48]. The choice of using ARs is based on practical reasons. It is easy to use and may have less impact on ordinary care work and less intrusion into participants’ integrity compared with video recordings [47], even though ARs could also be perceived as sensitive and uncomfortable. In this research, we will perform observations to study communication because of its advantages. At the same time, there is a potential risk that the participants will change their behavior or that the observations will disturb the usual practice. Second, additional challenges might arise from conducting stimulated recall with RNs due to a potential concern that RNs feel questioned about their communication and assessment during the observed visits. This could result in the tendency to formulate post-hoc explanations, refrain from disclosing negative aspects, or present responses that align with perceived social desirability [40]. Finally, in the present research, the data only include adults over 18 years of age. A large bulk of visits to primary healthcare is made by parents with small children. It is a limitation that our data do not include these consultations. Future research might also explore the triad of RN-child‒parent communication.

In conclusion, the PINPOINT project can contribute significantly to the development of clinically rooted methodologies for improving assessments and communication support by registered nurses in primary care (specific aim 5). To the best of our knowledge, this research will be the first-of-its kind to investigate practices of communication between patients and RNs and RNs’ decision-making to develop innovative and feasible solutions for successful and sustainable priority setting and to use a realist evaluation to examine its working mechanisms. To understand and help overcome challenges, this project will integrate an analysis of care needs and experiences as reported by patients with an analysis of communication during patient-RN visits and RNs’ experiences. Most likely, this research will contribute to knowledge that can strengthen RNs’ communication, assessments, and clinical decision-making in primary care. In the long term, this can contribute to how the competencies of different professionals are organized and carried out to make the best use of the resources within primary care service.

## Data Availability

The datasets generated and/or analyzed during the current study are not publicly available for ethical reasons but are available from the corresponding author upon reasonable request.

## List of abbreviations

AR: audio recordings
RN: registered nurse
VR-CoDES: the Verona Coding definition of emotional sequences
QUOTE-COMM: QUality Of care Through the patients’ Eyes
